# Multiple tumor marker protein chip detection system in diagnosis of pancreatic cancer

**DOI:** 10.1186/1477-7819-12-333

**Published:** 2014-11-08

**Authors:** Fangfeng Liu, Futian Du, Xiao Chen

**Affiliations:** Department of Hepatobiliary Surgery, Shandong Provincial Hospital Affiliated to Shandong University, 9677 Jingshi Road, Jinan, 250021 Shandong Province China; Department of Hepatobiliary Surgery, People’s Hospital of Weifang, Weifang, 261041 Shandong Province China

**Keywords:** Tumor marker, Protein biochip, Pancreatic cancer

## Abstract

**Background:**

The clinical stage of the disease at diagnosis often determines the prognosis and survival rate of a patient with pancreatic cancer. Early symptoms of pancreatic cancer are often not obvious on imaging (ultrasound, computed tomography (CT), and so on), and when patients present with weight loss, jaundice and abdominal pain and other symptoms, they are usually already in the advanced stages of pancreatic cancer. However, the examination of combined tumor markers might improve their sensitivity or specificity in aiding diagnosis.

**Methods:**

Twelve tumor markers including AFP, CEA, NSE, CA125, CA15-3, CA242, CA19-9, PSA, f-PSA, FER, β-HCG and HGH were measured by the protein biochip detection in serum in 235 pancreatic cancer patients, 230 benign pancreatic disease patients and 240 healthy people.

**Results:**

Positive detection rates of tumor markers were: CA19-9 (49.3%), CA125 (45.1%), FER (44.2%), CA242 (42.5%), CEA (38.6%), CA15-3 (36.7%), β-HCG (29.6%), AFP (24.5%), NSE (18.2%), PSA (19.5%), f-PSA (9.4%) and HGH (8.7%) respectively. There was significant difference in CA19-9, NSE, CEA, CA242 and CA125 by multi-tumor marker protein biochip detection among patients with cancer, benign disease and healthy people (*P* <0.05). The positive rate of 5 tumor markers was 94.9%, and this was much higher than that of any single marker.

**Conclusion:**

The detection of CA19-9, NSE, CEA, CA242 and CA125 in the multi-tumor marker protein biochip system is helpful in the diagnosis of pancreatic cancer.

## Background

Early detection of cancer has improved the survival of patients with many types of cancer and is critical for future improvements in effectively treating the disease [1]. The detection of serum tumor markers is an effective and non-invasive diagnostic or prognostic tool for pancreatic cancer. The two major reasons that most tumor markers are not used for tumor screening are their low sensitivity and specificity, resulting in low detection rates and unacceptable false-positive diagnoses [2]. The examination of combined tumor markers might improve the sensitivity or specificity [3].

The purpose of this study was to evaluate the diagnostic value of multi-tumor marker protein biochip detection for pancreatic cancer. This protein biochip system quantitatively measured 12 common tumor markers, including cancer antigen (CA)125, CA15-3, CA19-9, CA242, carcinoembryonic antigen (CEA), R-fetoprotein (AFP), prostate specific antigen (PSA), free-prostate specific antigen (f-PSA), human growth hormone (HGH), β-human chorionic gonadotropin (β-HCG), neuron-specific enolase (NSE) and ferritin (FER), in the serum and was tested in clinically confirmed cancer patients and apparently healthy individuals. The value of this biochip in cancer screening of apparently healthy populations and in cancer patients is discussed.

## Methods

### Patients and serum samples

From June 2012 to October 2013, 235 patients (150 men and 85 women, median age 61 years, range 20 to 78 years) with pancreatic cancer were included in the study group. One hundred and fifteen patients were treated with pancreaticoduodenectomy or distal pancreatectomy and 120 patients underwent a palliative operation. According to the *TNM Classification, fifth edition* [4], there were 22 stage I patients, 140 stage II patients and 73 stage III patients. The location of tumor was divided into head (48 cases), body/tail (146 cases) and the whole pancreas (59 cases). The tumor size was divided into equal to (12 cases) and smaller than 5 cm (154 cases), or larger than 5 cm (69 cases) in diameter. All diagnoses were confirmed by histology of postoperative or cytology of intraoperative biopsy examination. In addition, there were 230 benign pancreatic diseases (100 chronic pancreatitis and 130 benign adenomas, including 61 men and 39 women, age ranging from 20 to 82 years), and 240 blood donors and other volunteers known to be in good health.

### Sample preparation

Horseradish peroxidase (HRP) and chemiluminescence substrates (SuperSignal Femto maximum sensitivity) were purchased from Pierce (Rockford, IL, USA). Antigens and antibodies were produced in our laboratory. Antibody conjugation was done according to Nakane and Kawaio’s article [5].

### Sample measurement

**Figure 1:**
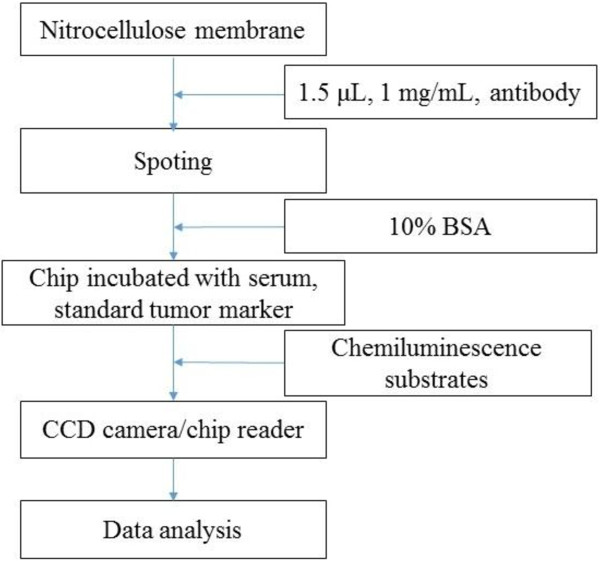
**The workflow of the chip detection system.**

One point five microliters of each of the 12 capture antibodies, with an average concentration of 1 mg/mL, were arrayed in duplicates on a 1 × 1 cm nitrocellulose membrane. The array consisted of a 5 × 5 matrix with 12 pairs of antibody spots and 1 blank spot as control. After spotting, the membrane was mounted with a plastic mold and blocked with 10% BSA. Each protein chip was incubated with 100 μL of serum, rinsed and washed with TBST (0.1 mol Tris-HCl, 8.5 g NaCl, 1 mL Tween 20, pH 7.6). The chip was then incubated with 100 μL of HRP-labeled antibodies for 30 minutes at 37°C and again rinsed and washed with TBST. Chemiluminescence substrates were added for 1 minute. Light signals were captured with the self-built chip reader based on a CCD camera and controlled with a computer system using self-developed software. The workflow of the chip detection system is shown in Figure 1.

### Statistical analysis

Due to non-normal distribution of the raw data, a logarithmic transformation was needed. Data collected were subjected to appropriate transformation (square root) before analysis of variance was performed and means were separated by SAS-SNK (*P* <0.05) test (SAS Institute, Cary, NC, USA; 1989).

## Results

Positive detection rates of tumor markers were: CA19-9 (49.3%), CA125 (45.1%), FER (44.2%), CA242 (42.5%), CEA (38.6%), CA15-3 (36.7%), β-HCG (29.6%), AFP (24.5%), NSE (18.2%), PSA (19.5%), f-PSA (9.4%) and HGH (8.7%) respectively (Table 1).

**Table 1:** **Twelve (C-12) protein biochip test results of healthy, benign pancreatic and pancreatic cancer subjects**

	Healthy	Benign pancreatic	Pancreatic cancer	***P***-value
	(n =240)	(n =230)	(n =235)	(SNK method)
CA19-9 (U/ml)	4.5 ± 2. 8	16.4 ± 19.2	203.8 ± 296.5	0. 0008
CA125 (U/ml)	2.9 ± 2. 6	18.9 ± 28.7	174.6 ± 266.2	0. 0016
FER (ng/ml)	118.9 ± 62.6	320.9 ± 285	373.8 ± 385.5	0. 5579
CA242 (U/ml)	2.3 ± 0.8	9.3 ± 14.7	130.5 ± 150.5	0. 0035
CEA (ng/ml)	1.1 ± 0.5	1.87 ± 2.3	13.5 ± 27.1	0. 0392
CA15-3 (U/ml)	5.1 ± 4.2	13.4 ± 6.8	79.4 ± 82.3	0. 0584
β-HCG (mIU/ml)	0.1 ± 0.1	0.5 ± 0.4	8.96 ± 12.8	0. 0694
AFP (ng/ml)	3.3 ± 2.4	1.6 ± 1.8	14.5 ± 36.8	0. 0568
NSE (ng/ml)	1.3 ± 0.8	2.1 ± 2.3	7.9 ± 12.8	0. 0179
PSA (ng/ml)	0.3 ± 0.2	0.6 ± 0.5	1.5 ± 2.38	0. 0575
f-PSA (ng/ml)	0.1 ± 0.1	0.2 ± 0.2	0.6 ± 0.6	0. 1046
HGH (ng/ml)	0.1 ± 0.2	0.8 ± 0.7	4.5 ± 4.8	0. 0564

**Figure 2:**
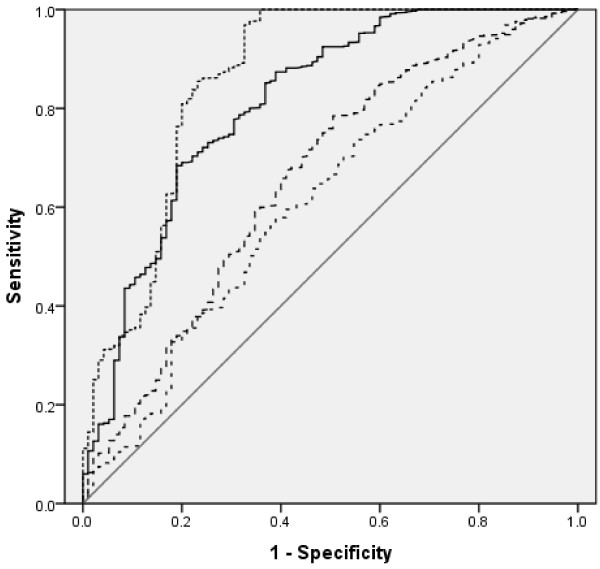
**ROC curve.**

There was significant difference in CA19-9, NSE, CEA, CA242, CA125 by multi-tumor marker protein biochip detection among patients with cancer, benign disease and apparently healthy people (*P* <0.05). We took the normal value of tumor marker serum level as cut-off value to determine the negative or positive likelihood of pancreatic cancer. The positive rate of 5 tumor markers was 94.9%, which was much higher than that of any single marker (Table 2). Figure 2 shows the receiver operating characteristic (ROC) curves of the 5 markers. The diagnostic performance of markers was ranked according to the area under the curves.

**Table 2:** **Positive rate of pancreatic cancer versus benign pancreatic disease**

	Pancreatic cancer	Benign pancreatic disease
	(n =235)	(n =230)
CA19-9 (U/ml)	49.3%	14.2%
CA125 (U/ml)	47.3%	13.6%
FER (ng/ml)	43.2%	49.2%
CA242 (U/ml)	42.7%	23.6%
CEA (ng/ml)	36.4%	14.8%
CA15-3 (U/ml)	28.7%	0
β-HCG (mIU/ml)	25.5%	0
AFP (ng/ml)	20.4%	0
NSE (ng/ml)	18.9%	0
PSA (ng/ml)	13.5%	0
f-PSA (ng/ml)	14.1%	0

Source of the curve: —— The combined five markers, - - - CEA, · · · CA125, · · · CA15-3, —— Reference line.

It was noticed that the CA19-9 levels were significantly higher in patients with cancer of the pancreatic body and tail than of the pancreatic head (*P* =0.047). There was no correlation of serum CEA and CA242 with tumor location (*P* >0.05). The serum levels of CEA, CA19-9, and CA242 were obviously higher in stage III patients than in stages I and II (*P* <0.05). However, the serum levels of CEA, CA19-9 and CA242 in patients with pancreatic cancer were not affected by the tumor size (*P* >0.05) (Table 3).

**Table 3:** **The relationship between the serum markers and the location, size, and TNM stage of pancreatic cancer**

Group	Number	CEA		CA19-9		CA242	
		value (ng/ml)	***P***-value	value (ng/ml)	***P***-value	value (ng/ml)	***P***-value
Tumor location head	48	20.6 ± 42.6		756.6 ± 1,228.2		58.3 ± 55.3	
Body/tail	146	28.0 ± 33.1		2,902.5 ± 3,308.3		76.1 ± 72.1	
Whole	59	74.9 ± 232.1	*P* >0.5	1,517.3 ± 2,928.6	*P* <0.5	72.6 ± 60.3	*P* >0.5
Tumor size							
≤ 5 cm	166	23.7 ± 39.1		1,113.4 ± 2,660.4		45.2 ± 48.1	
> 5 cm	69	23.9 ± 36.3	*P* >0.5	1,258.9 ± 5,002.3	*P* >0.5	59.1 ± 60.3	*P* >0.5
TNM stage							
I	22	14.1 ± 16.6		1,126.6 ± 1,425.9		57.6 ± 54.4	
II	140	12.7 ± 28.3		845.0 ± 2,000.7		53.9 ± 52.1	
III	73	90.3 ± 212.8	*P* <0.5	2,976.6 ± 3,513.2	*P* <0.5	97.6 ± 67.1	*P* <0.5

## Discussion

Due to their limited specificity, the measurement of a single tumor marker is usually not sufficient to diagnose cancer. Impressive integration efforts are demonstrated by the ability to perform on-chip trypsin digestion, separation and injection into a mass spectrometer with a single device [6]. Elevated levels of the proteins CEA, AFP, hCG-β, FER, CA15-3, CA19-9, and CA125 can be associated with lung, pancreatic, breast, colorectal, and other types of cancer [7–10]. Several studies have shown that the measurement of panels of tumor markers can improve their diagnostic value [5–8]. In previous reports, the levels of serum CEA, CA19-9 and CA242 in patients with pancreatic cancer were higher than those of other malignant diseases and benign pancreatic diseases [7, 11]. Here, we found that the combination of CEA and CA242 could increase the specificity to 94.9% in the diagnosis of pancreatic cancer. This is important in helping to differentiate pancreatic cancer from other malignancies and benign pancreatic diseases.

## Conclusion

The biosensor system described here is suitable for the measurement of a wide range of biomarkers. We compared the serum levels of CA19-9, CA125, FER, CA242, CEA, CA15-3, β-HCG, AFP, NSE, PSA, f-PSA and HGH associated with pancreatic cancer, and found that simultaneous analysis of them was important for the diagnosis of pancreatic cancer. The detection of CA19-9, NSE, CEA, CA242 and CA125 in the multi-tumor markers protein biochip system is helpful in the diagnosis of pancreatic cancer.

### Statement

The study was approved by the local ethical committee and all individuals provided written informed consent for study participation.

## Abbreviations

CT: computed tomography
ROC: Receiver operating characteristic
CA15-3: carbohydrate antigen 15-3
β-HCG: β-human chorionic gonadotrophin
AFP: α-fetoprotein
NSE: 2-phospho-D-glycerate hydrolase
PSA: prostate specific antigen
f-PSA: free-prostate specific antigen
HGH: human Growth Hormone.

## References

[1] Hollingsworth MA (2013). Translational Implications of Molecular Genetics for Early Diagnosis of Pancreatic Cancer.

[2] Sun Z, Fu X, Zhang L, Yang X, Liu F, Hu G (2004). A protein chip system for parallel analysis of multi-tumor markers and its application in cancer detection. Anticancer Res.

[3] Zhao XY, Yu SY, Da SP, Dai L, Guo XZ, Dai XJ, Wang YM (1998). A clinical evaluation of serological diagnosis for pancreatic cancer. World J Gastroenterol.

[4] Sobin LH, Fleming ID (1997). TNM classification of malignant tumors. Cancer.

[5] Nakane PK, Kawaoi A (1974). Peroxidase-labeled antibody a new method of conjugation. J Histochem Cytochem.

[6] Mouradian S (2002). Lab-on-a-chip: applications in proteomics. Curr Opin Chem Biol.

[7] Carpelan-Holmstrom M, Louhimo J, Stenman UH, Alfthan H, Haglund C (2001). CEA, CA 19-9 and CA 72-4 improve the diagnostic accuracy in gastrointestinal cancers. Anticancer Res.

[8] Louhimo J, Finne P, Alfthan H, Stenman UH, Haglund C (2002). Serum HCGβ, CA 72-4 and CEA are independent prognostic factors in colorectal cancer. Int J Cancer.

[9] Hayakawa T, Naruse S, Kitagawa M, Ishiguro H, Kondo T, Kurimoto K, Fukushima M, Takayama T, Horiguchi Y, Kuno N, Noda A, Furukawa T (1999). A prospective multicenter trial evaluating diagnostic validity of multivariate analysis and individual serum marker in differential diagnosis of pancreatic cancer from benign pancreatic diseases. Int J Pancreatol.

[10] Wilson MS, Nie W (2006). Multiplex measurement of seven tumor markers using an electrochemical protein chip. Anal Chem.

[11] Haglund C (1986). Tumour marker antigen CA125 in pancreatic cancer: a comparison with CA19-9 and CEA. Br J Cancer.

